# Establishment, molecular and biological characterization of HCB-514: a novel human cervical cancer cell line

**DOI:** 10.1038/s41598-018-38315-7

**Published:** 2019-02-13

**Authors:** Marcela Nunes Rosa, Adriane Feijó Evangelista, Letícia Ferro Leal, Cristina Mendes De Oliveira, Viviane Aline Oliveira Silva, Carla Carolina Munari, Fernanda Franco Munari, Graziela De Macêdo Matsushita, Ricardo Dos Reis, Carlos Eduardo Andrade, Cristiano de Pádua Souza, Rui Manuel Reis

**Affiliations:** 1 Molecular Oncology Research Center, Barretos Cancer Hospital, Barretos, SP Brazil; 2 Department of Pathology, Barretos Cancer Hospital, Barretos, SP Brazil; 3 Department of Gynecologic Oncology, Barretos Cancer Hospital, Barretos, SP Brazil; 4 Life and Health Sciences Research Institute (ICVS), School of Health Sciences, University of Minho, Braga, Portugal; 5 3B’s—PT Government Associate Laboratory, Braga/Guimarães, Portugal

## Abstract

Cervical cancer is the fourth most common cancer in women. Although cure rates are high for early stage disease, clinical outcomes for advanced, metastatic, or recurrent disease remain poor. To change this panorama, a deeper understanding of cervical cancer biology and novel study models are needed. Immortalized human cancer cell lines such as HeLa constitute crucial scientific tools, but there are few other cervical cancer cell lines available, limiting our understanding of a disease known for its molecular heterogeneity. This study aimed to establish novel cervical cancer cell lines derived from Brazilian patients. We successfully established one (HCB-514) out of 35 cervical tumors biopsied. We confirmed the phenotype of HCB-514 by verifying its’ epithelial and tumor origin through cytokeratins, EpCAM and p16 staining. It was also HPV-16 positive. Whole-exome sequencing (WES) showed relevant somatic mutations in several genes including *BRCA2*, *TGFBR1* and *IRX2*. A copy number variation (CNV) analysis by nanostring and WES revealed amplification of genes mainly related to kinases proteins involved in proliferation, migration and cell differentiation, such as *EGFR*, *PIK3CA*, and *MAPK7*. Overexpression of EGFR was confirmed by phospho RTK-array and validated by western blot analysis. Furthermore, the HCB-514 cell line was sensitive to cisplatin. In summary, this novel Brazilian cervical cancer cell line exhibits relevant key molecular features and constitutes a new biological model for pre-clinical studies.

## Introduction

Cervical cancer is a major public health problem worldwide, making it the fourth most common type of cancer among women. In 2018, there were 570,000 new cases reported and 311,000 related deaths^1^. Women between the ages of 50 and 60 years-old are most affected by cervical cancer^2^. Persistent infection of the basal layer of cervical epithelium with high-risk human papillomavirus (HPV), such as 16, 18, 31, 33, 35, 39, 45, 51, 52, 56, 58, 59 and 68, is considered the main risk factor for the development of cervical cancer precursors, known as cervical intraepithelial neoplasia (CIN 1, 2, and 3), and invasive cervical cancer^3^.

Cervical cancer can be histologically classified as squamous cell carcinoma (SCC), adenocarcinoma and adenosquamous carcinoma, with a prevalence of 77%, 17% and 6%, respectively^4^. Many efforts have being undertaken to determine the molecular profile of this heterogeneous disease. Recently, The Cancer Genome Atlas (TCGA) integrated information from CNV, methylation, mRNA, and miRNA profiles. Through clustering, the TCGA revealed three molecular subtypes of cervical cancer: SCC keratin-high, SCC keratin-low and adenocarcinoma. Differences observed in the three major subtypes included the enriched expression of some genes such as *PIK3CA*, *ADH7* and *SPRR3* in the SCC keratin-high compared with the SCC keratin-low cluster; more frequent CNVs including common EGFR amplifications in SCCs; a high number of aberrations in tumor-suppressor genes related with TGF-β pathway in adenocarcinomas including *SMAD4* and *TGFBR2* deletions, and increased DNA methylation in adenocarcinomas^4,5^.

Cervical cancer treatment is based on the stage of disease. For early stage disease, surgery is the primary treatment modality, cure rates are high, and 5-year overall survival is up to 92%^6^. For advanced disease, which includes recurrent or metastatic disease, the mainstay of therapy is chemoradiation with a platinum-based agent and unfortunately, treatment responses are poor^7^. To improve outcomes for patients with advanced disease, recent findings on the molecular profile of this tumor type is valuable.

To facilitate the discovery of new antineoplastic agents, many research centers and teams have been carrying out screenings with a multitude of compounds, testing them in *in vitro* models, using immortalized human cancer cell lines^8^. This approach provides controlled conditions to evaluate the efficacy of drugs, and enables the unrestricted availability of human source material. However, there is a very low number of cervical cancer cell lines commercially available in comparison with other tumors, such as breast and lung tumors, which currently provides a limited representation of known subtypes and tumor heterogeneity. Therefore, the aim of this study was to establish and to characterize a new human cervical tumor cell line derived from a Brazilian patient.

## Results

### Clinical characterization and establishment of a primary cell culture

**Figure 1:**
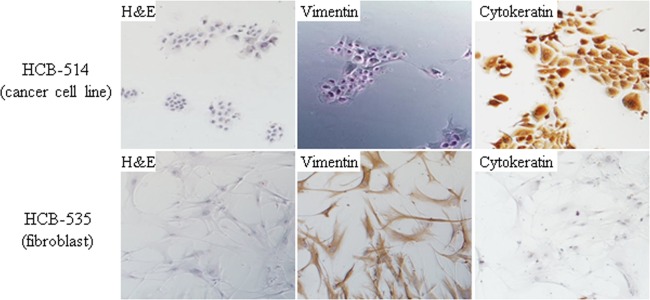
Representative images of immunocytochemistry of cervical cancer cell line HCB-514 (top images) and the fibroblast cells (HCB-535) (bottom images). All pictures were taken at 100x magnification.

From March 2016 to June 2017, 35 cervical tumor biopsies were processed (Suppl. Table 1). Only one (2,9%) of the cell cultures, named HCB-514, survived for more than 12 months and continued to grow after several freeze-thaw cycles. This cell line was derived from a 30 year-old patient diagnosed with stage IIB squamous cell carcinoma of the cervix. The patient was treated with concurrent chemoradiation with cisplatin from October 10 to November 17, 2016, and was disease-free through her most recent follow-up appointment, on April 25, 2018. The cell culture HCB-514 grew attached to the flask, with cells forming an irregular island pattern with a cobblestone morphology, characteristic of epithelial cells (Fig. 1). When the cell line became confluent, cells were frozen in 5% DMSO in fetal bovine serum (FBS) solution in liquid nitrogen for further assays. After the fourth passage, immunophenotypic characterization was performed. The HCB-514 cell line presented stable outgrowing for more than 6 months, reaching 26 passages, and it was HPV-positive, supporting a spontaneous immortalization process. The cell line was negative for mycoplasma, and a short tandem repeat (STR) analysis showed that the HCB-514 cell line, tumor tissue and peripheral blood shared the same markers, confirming cell line identity (Table 1).

**Table 1:** STR profile of cell culture, blood and frozen tissue of the patient.

Regions	Blood	Tumor	HCB-514
Amelogenin	X	X	X
CSF1PO	10,12	10,12	12
D13S317	9,11	9,11	9,11
D16S539	9	9	9
D5S818	10,12	10,12	10
D7S820	11,13	11,13	13
THO1	7,9	7,9	7,9
TPOX	8,12	8,12	8,12
WA	16	16	16

### Immunophenotypic characterization

**Figure 2:**
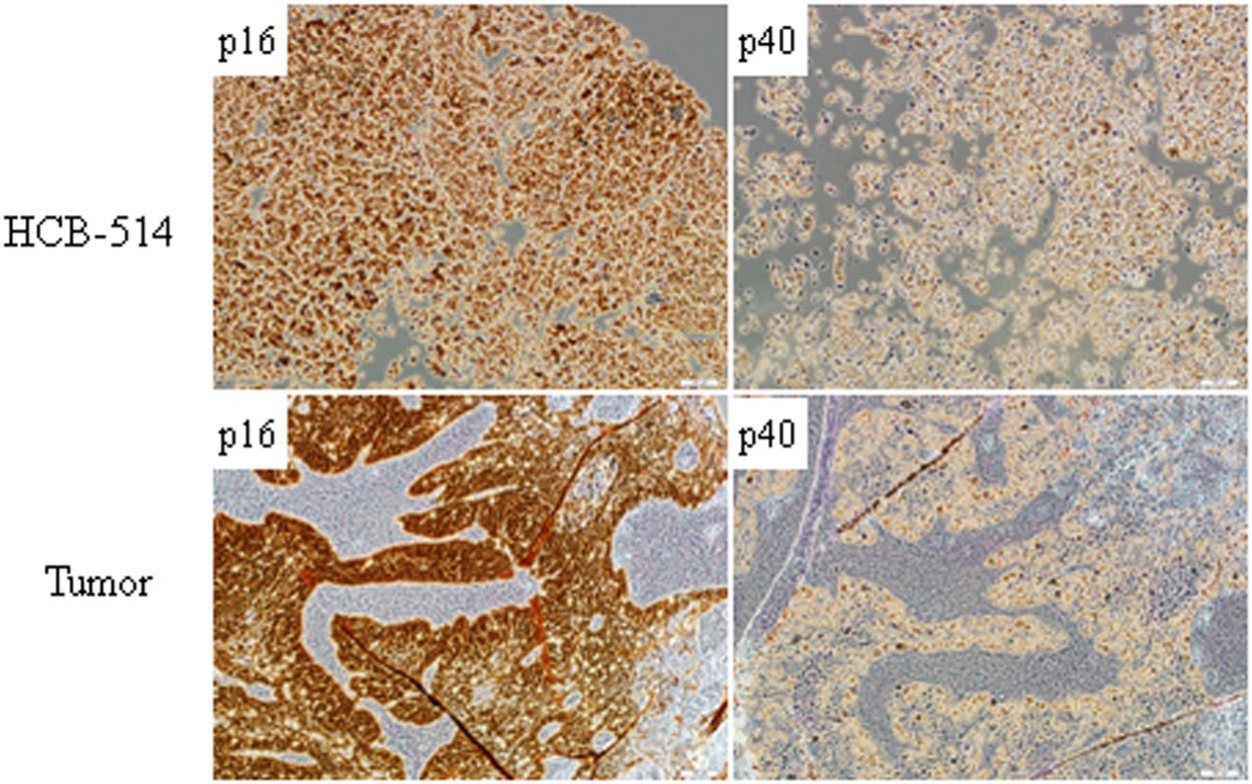
Representative immunohistochemistry images of HCB-514 cell block slices (top images) and the tumor patient tissue (bottom images) depicting expression of p16 (cervical cancer marker) and p40 (squamous cell carcinoma marker). All pictures were taken at 100x magnification.

Immunocytochemistry of HCB-514 showed strong cytokeratin expression, but no vimentin expression, similar to SiHa, indicating that HCB-514 is an epithelial cell line (Fig. 1). On the other hand, HCB-535 fibroblast presented the opposite staining pattern, with strong vimentin expression but no cytokeratin expression (Fig. 1). Furthermore, immunohistochemistry from HCB-514 cell block was in accordance with the expression pattern of formalin-fixed paraffin embedded (FFPE) tumor tissue taken in parallel from the patient at the time of biopsy. Both HCB-514 and FFPE tumor tissue were positive for p40, a squamous cell marker, and p16, a marker for cervical cancer (Fig. 2), confirming its squamous cell origin.

**Figure 3:**
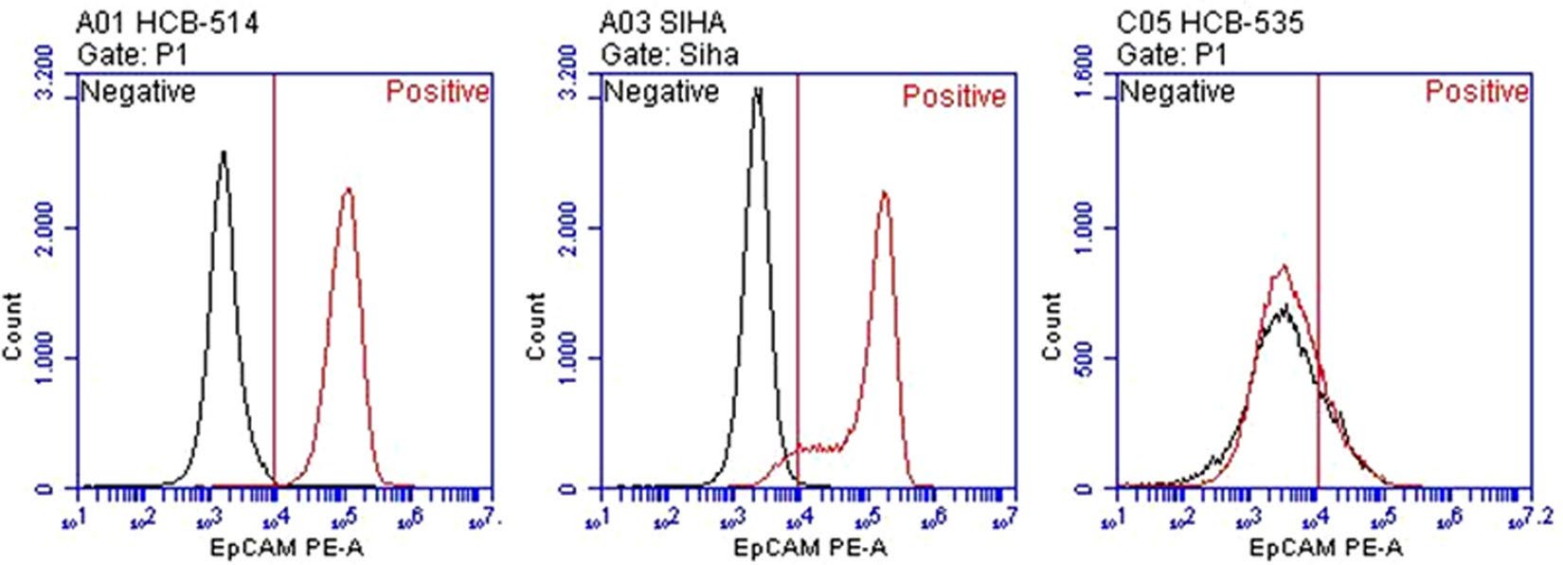
Evaluation of epithelial cellular adhesion molecule (EpCAM) expression in HCB-514. HCB-514 (left), SiHa (center) and HCB-535 (right) cell lines were stained with antibody anti-EpCAM and evaluated in flow cytometry. SiHa, and a fibroblast cell line (HCB-535) were used as positive and negative controls, respectively. Black graphs represent unstained cells; red graphs represent stained cells.

In addition, flow cytometry analysis showed high expression of EpCAM in HCB-514, with 100% of cells staining positive. Expression of EpCAM was present in 92% of SiHa cells and in 19% of the fibroblast cell line (Fig. 3).

### Doubling-time

**Figure 4:**
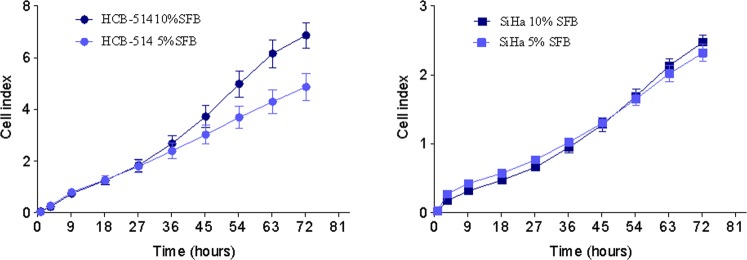
Growth curves of HCB-514 obtained from real time impedance-based technology cell analyzer system (xCELLigence). Different media conditions were assessed. Data represent the mean of 3 independent experiments done in duplicate.

The growth curve for HCB-514 during exponential cell growth showed a doubling time of 18 h in 10% FBS media, and 24 h in 5% FBS media. To evaluate whether this growth was faster or slower than other cell lines largely used for *in vitro* assays, SiHa was also evaluated and showed a doubling-time of 17 h in 10% FBS media and 21 h in 5% FBS. Thus, the time was similar among cell lines, with a faster doubling-time in 10% than in 5% media (Fig. 4).

### HPV status and genotyping

HPV infection is present in almost all cervical tumors, therefore we evaluated the presence of the virus in the HCB-514 cell line. For this purpose, GP5+/GP6+ primers were used to amplify the highly conserved region of the HPV L1 gene by PCR. The band correspondent to this region was found in HCB-514, confirming the presence of HPV (Suppl. Fig. 1a). To identify which high-risk HPV type was present, a genotyping test was performed with the COBAS® HPV assay, confirming HPV type 16. Furthermore, to confirm that HPV16 infection was present, we evaluated and confirmed the presence of E6 oncoprotein, an important protein responsible for HPV oncogenicity (Suppl. Fig. 1b).

Furthermore, WES allowed us to determine whether the HPV genome integrated into host genomic DNA of HCB-514 and in primary tumor. The results showed two integration sites, one into the *MICU2* and the other into *CDH13* genes, the last being also found in the WES of patient’s primary tumor.

Moreover, it is hypothesized that HPV genetic variation co-evolves with hosts of certain ethnic groups, which influences disease development and outcome^8^. Therefore, to identify the variant of HPV in HCB-514 and in the primary cervical cancer, phylogenetic analysis was performed. Both the HCB-514 cell line and the patient’s primary tumor showed the presence of the sublineage HPV16 A1 (Suppl. Fig. 2).

### HCB-514 and tumor mutational profile

The WES analysis of paired cell line/blood revealed 520 somatic mutations. The analysis of the most significant non-silent mutations identified 93 mutated genes (Suppl. Table 3), such as *HPS-3*, *IRX2*, *XPO5*, *CBWD1*, *TGFBR1*, *CUBN*, *TRHDE*, *BRCA2*, *THBS1*, *TGM5*, *CCDC22*, which were previously described as pathogenic, or likely pathogenic in tumors (Suppl. Table 3).

Regarding the analysis of paired primary tumor/blood, 863 mutations were found, with 68 non-silent mutations identified (Suppl. Table 4). Among them, 18 mutations were shared with HCB-514 (Table 2), including mutations in the *BRCA2*, *TGFBR1*, *CCDC22* and *SHKBP1* genes.

**Table 2:** Somatic non-silent mutations found in both HCB-514 and tumor.

Chromosome	Position	Gene	Referrence allele	Variant allele	Aminoacid	Zygosity
1	228335177	GUK1	C	T	p.R129W	Heterozygous
2	69049563	ARHGAP25	G	A	p.G431E	Heterozygous
5	140793112	PCDHGA10	G	A	p.E124K	Heterozygous
7	77408339	RSBN1L	A	C	p.K799Q	Heterozygous
9	101900171	TGFBR1	C	T	p.A202V	Heterozygous
10	51130414	PARG	G	A	p.L59F	Heterozygous
12	99194813	ANKS1B	C	G	p.E1053Q	Heterozygous
12	109201563	SSH1	G	A	p.R193W	Heterozygous
13	32945095	BRCA2	G	A	p.W2830*	Heterozygous
15	39874463	THBS1	G	A	p.R46H	Heterozygous
15	43545745	TGM5	G	A	p.R215W	Heterozygous
19	41084418	SHKBP1	G	A	p.R129W	Heterozygous
20	4163484	SMOX	G	A	p.G431E	Heterozygous
20	23066708	CD93	G	A	p.E124K	Heterozygous
21	47421214	COL6A1	G	C	p.K799Q	Heterozygous
22	17590484	IL17RA	C	G	p.A202V	Heterozygous
22	19213831	CLTCL1	C	T	p.L59F	Heterozygous
X	49105196	CCDC22	G	T	p.E1053Q	Heterozygous

Several studies have reinforced the putative impact of APOBEC family of cytidine deaminases enzymes in altered nucleotides found in cervical cancer^9^. Therefore, mutational signatures from cell line and tumor were analyzed. Both samples showed similar mutation signatures, with C to A and C to T being the most predominant (Suppl. Fig. 3).

**Figure 5:**
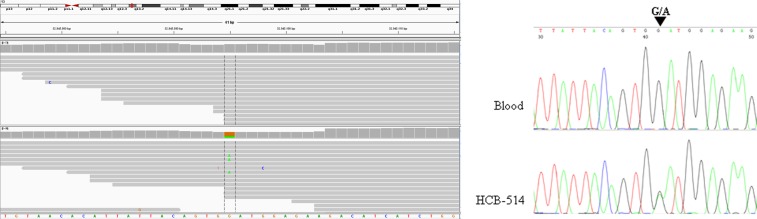
IGV image from the whole-exome sequencing (WES) result of *BRCA2* gene (left) and electropherogram of exon 20 from *BRCA2* gene that harbors mutation (G → A) (right).

The *BRCA2* mutation (p.W2830*) identified was further confirmed by Sanger sequencing (Fig. 5).

### Copy number variation

Nanostring analysis for CNV was performed for both the cell line and constitutive DNA. Among all evaluated genes, 44 somatic alterations were discovered, including 30 regions with amplification, containing genes such as *MYC*, *TP53*, *EGFR*, *TERT*, *AKT3*, *MAPK7*, *BIRC2*, *YAP1* and 14 losses, containing genes including *KIT*, *RB1*, *BRCA2* and *APC* (Tables 3 and 4).

**Table 3:** Copy number amplification regions in HCB-514 cell line.

Chromosome Region	CNV	Cytoband	Cancer genes
chr1	Amplification	p36.33; q32.1	
chr1	High amplification	q32.1 - q43	*H3F3A*, *FH*
chr1	Amplification	q43 - q44	*AKT3*
chr2	High amplification	p25.3 - p22.3	*MYCN*, *C2orf44*, *NCOA1*, *DNMT3A*, *ALK*
chr2	High amplification	p22.3 - p21	*EML4*
chr2	Amplification	p21 - q33.1	*MSH2*, *MSH6*, *FBXO11*, *BCL11A*, *REL*, *XPO1*, *IGL@*, *TTL*, *PAX8*, *ERCC3*, *CHN1*, *HOXD13*, *HOXD11*, *NFE2L2*, *PMS1*, *SF3B1*
chr2	Amplification	q37.3	
chr3	High amplification	q11.1 - q26.1	*TFG*, *CBLB*, *GATA2*, *RPN1*, *FOXL2*, *WWTR1*, *GMPS*, *MLF1*
chr3	High amplification	q26.1 - q29	*EVI1*, *PIK3CA*, *SOX2*, *ETV5*, *EIF4A2*, *BCL6*, *LPP*, *DCUN1D1*, *PRKCI*
chr3	High amplification	q29	*TFRC*
chr5	Amplification	p15.33 - p11	*IL7R*, *LIFR*, *SKP2*, *TERT*
chr6	Amplification	q12	
chr7	Amplification	p22.3 - q11.21	*CARD11*, *PMS2*, *ETV1*, *HNRNPA2B1*, *HOXA9*, *HOXA11*, *HOXA13*, *JAZF1*, *IKZF1*, *EGFR*
chr8	Amplification	p23.3 - q24.3	*PCM1*, *WRN*, *WHSC1L1*, *FGFR1*, *HOOK3*, *TCEA1*, *PLAG1*, *CHCHD7*, *NCOA2*, *HEY1*, *COX6C*, *EXT1*, *MYC*, *NDRG1*, *RECQL4*, *C8orf33*, *C8orf4*
chr9	Amplification	q32	
chr11	Amplification	q12.3 - q13.4	*MEN1*, *CCND1*, *ORAOV1*, *FADD*
chr11	Amplification	q13.4 - q22.1	*NUMA1*, *PICALM*, *MAML2*, *GAB2*, *YAP1*
chr11	High amplification	q22.1 - q22.3	*BIRC2*, *BIRC3*
chr12	Amplification	p13.33	
chr12	Amplification	q23.1 - q24.33	*ALDH2*, *PTPN11*, *BCL7A*
chr14	Amplification	q11.1 - q32.33	*BCL2L2*, *CCNB1IP1*, *TRA@*, *NKX2-1*, *NIN*, *KTN1*, *GPHN*, *TSHR*, *TRIP11*, *GOLGA5*, *DICER1*, *TCL6*, *TCL1A*, *BCL11B*, *AKT1*, *IGH@*
chr15	Amplification	q21.3 - q26.3	*IGF1R*, *FLJ27352*, *TCF12*, *PML*, *NTRK3*, *IDH2*, *CRTC3*, *BLM*
chr16	Amplification	p13.3	
chr16	High amplification	p13.3 - p11.2	*TSC2*, *CREBBP*, *CIITA*, *SOCS1*, *TNFRSF17*, *ERCC4*, *MYH11*, *PALB2*, *IL21R*, *FUS*
chr17	Amplification	p13.3 - q11.2	*MAPK7*, *YWHAE*, *USP6*, *TP53*, *PER1*, *GAS7*, *MAP2K4*, *NF1*, *SUZ12*
chr18	High amplification	p11.31	
chr18	Amplification	p11.31 - p11.23	
chr18	Amplification	p11.1 - q23	*DCC*, *ZNF521*, *SS18*, *MALT1*, *BCL2*
chr19	Amplification	p13.2	
chr20	Amplification	p13 - q13.33	*BCL2L1*, *NCOA3*, *AURKA*, *ZNF217*, *EEF1A2*, *ASXL1*, *MAFB*, *TOP1*, *SDC4*, *GNAS*, *SS18L1*

**Table 4:** Copy number deletion regions in HCB-514 cell line.

Chromosome Region	Event	Cytoband	Cancer genes
chr1	Deletion	p11.2 - q21.1	
chr2	Deletion	q33.1 - q37.3	*CREB1*, *IDH1*, *ATIC*, *FEV*, *PAX3*, *ACSL3*
chr3	Deletion	p26.3 - p12.3	*SRGAP3*, *FANCD2*, *VHL*, *PPARG*, *RAF1*, *XPC*, *MLH1*, *MYD88*, *CTNNB1*, *SETD2*, *BAP1*, *PBRM1*, *FHIT*, *MITF*, *FOXP1*
chr3	Deletion	q26.1	
chr4	Deletion	p16.3 - p16.1	*FGFR3*, *WHSC1*
chr4	Deletion	p16.1 - q22.1	*SLC34A2*, *PHOX2B*, *FIP1L1*, *PDGFRA*, *CHIC2*, *KIT*, *KDR*
chr4	Homozygous deletion	q22.1	
chr4	Deletion	q22.1 - q26	*RAP1GDS1*, *TET2*
chr4	Deletion	q26 - q35.2	*IL2*, *FBXW7*
chr5	Deletion	p15.33	
chr5	Deletion	q11.1 - q13.2	*IL6ST*, *PIK3R1*
chr5	Homozygous deletion	q13.2	
chr5	Deletion	q13.2 - q31.3	*APC*
chr5	Deletion	q31.3 - q35.3	*PDGFRB*, *CD74*, *ITK*, *EBF1*, *RANBP17*, *TLX3*, *NPM1*, *NSD1*
chr7	Deletion	q11.21 - q22.1	*SBDS*, *ELN*, *HIP1*, *AKAP9*, *CDK6*
chr7	Homozygous deletion	q22.1	
chr7	Deletion	q22.1 - q35	*MET*, *SMO*, *CREB3L2*, *KIAA1549*, *BRAF*
chr7	Deletion	q35 - q36.3	*EZH2*, *MLL3*, *SHH*
chr9	Homozygous deletion	p13.2 - p13.1	
chr9	Deletion	p13.1 - p12	
chr9	Homozygous deletion	p12 - p11.2	
chr9	Deletion	p11.2 - q13	
chr9	Deletion	q13	
chr9	Deletion	q21.11	
chr9	Deletion	q21.32	
chr11	Deletion	q22.3 - q25	*ATM*, *DDX10*, *POU2AF1*, *SDHD*, *PAFAH1B2*, *PCSK7*, *MLL*, *DDX6*, *CBL*, *ARHGEF12*, *FLI1*
chr13	Deletion	q11 - q34	*CDX2*, *FLT3*, *BRCA2*, *LHFP*, *LCP1*, *RB1*, *ERCC5*, *FOXO1*, *GPC5*, *IRS2*
chr18	Deletion	p11.32 - p11.31	
chr18	Deletion	p11.23 - p11.21	
chr18	Deletion	p11.21	
chr19	Deletion	q13.42	
chr21	Deletion	q11.2 - q22.12	*OLIG2*, *RUNX1*
chr21	Deletion	q22.2 - q22.3	*TMPRSS2*, *U2AF1*
chrX	Deletion	q24	

To expand the analysis beyond the 87 genes of the NanoString panel, CNV was also investigated from WES data. The WES showed 64 chromosomal regions harboring a large number of genes with CNV. There were 30 amplified regions, with 9 of them presenting high amplification, harboring genes such as *MYNC*, *ALK*, *PIK3CA*, *BIRC3*, *ERCC4* and *PALB2* (Table 3). Deletions were found in 34 regions, harboring genes such as *MLH1*, *PDGFRA*, *APC*, *MET* and *RB1*. Homozygous deletions were found in 5 regions (Table 4).

Many of the amplified genes identified are related with PI3K-AKT and MAPK signaling pathways. Other altered regions harbored genes mostly related with the DNA repair process, apoptosis and transcriptional factors. Moreover, the cell line presented alterations in tyrosine-kinase receptors, such as *EGFR* and *ALK* amplification and *PDGFRA* and *MET* deletion.

**Figure 6:**
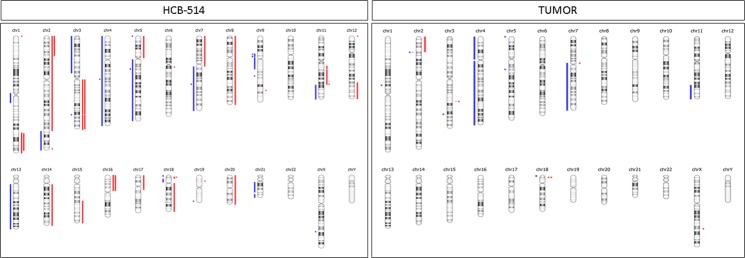
Copy number variations from whole-exome sequencing (WES) of HCB-514 (left) and tumor (right) were compared with patient’s blood DNA. Amplifications are shown in red markers and deletions in blue markers.

Regarding the CNV present in patient’s primary tumor, the number of CNVs was reduced compared with the HCB-514 cell line, with 4 amplification regions and 10 deletion regions. Again, both primary tumor and cell line shared some of these alterations, such as *MYCN* and *ALK* amplification and *PDGFRA*, *KIT*, *IL2*, *FBXW7*, *MET* and *ATM* deletion (Suppl. Table 4). Figure 6 summarizes CNV in the HCB-514 cell line and primary tumor.

### Phospho-RTK expression

**Figure 7:**
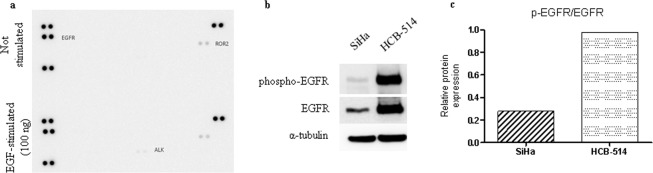
(**a**) Phospho-RTK arrays of HCB-514 were done at basal conditions (up) and upon EGF-stimulation (down). Each RTK is represented in duplicate in the arrays (two spots side-by-side), and three pairs of phospho-tyrosine positive controls are located in the corners of each array. (**b**) *Western blot* of EGFR phosphorylation, showing the different amount of phospho-EGFR and total EGFR compared to the SiHa cell line. Alpha-tubulin was used as endogenous control. (**c**) Quantification of phospho-EGFR normalized with total EGFR.

The characterization of RTK expression in HCB-514 revealed basal protein levels of activated EGFR and ROR2 (Fig. 7a, top). Upon EGF stimulation, the only new receptor activated was ALK (Fig. 7a, bottom). Activation of EGFR was also confirmed by the presence of phosphorylated EGFR in *Western blot* analysis (Fig. 7b), with 3.5-fold more expression in HCB-514 than in SiHa (Fig. 7c).

### Response to Cisplatin

**Figure 8:**
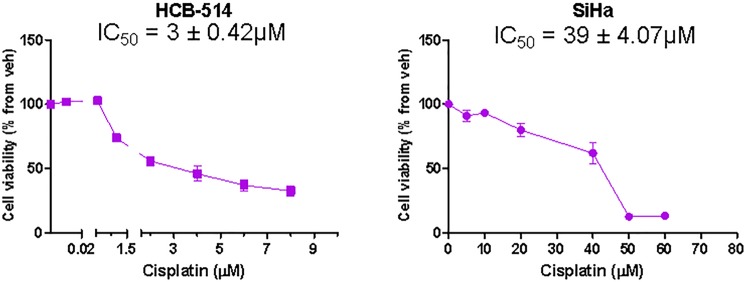
Cell viability of HCB-514 and SiHa after 72 h of cisplatin treatment. Results were obtained using the MTS assay. Data represent a mean of 3 independent experiments done in triplicate.

To evaluate the sensitivity of HCB-514 to the chemotherapeutic agent cisplatin, commonly used in clinical practice for cervical cancer treatment, HCB-514 was treated for 72 hours and a MTS assay was performed. The response to cisplatin was dose-dependent and the inhibitory concentration for 50% cells (IC_50_) value for HCB-514 was 3 ± 0.42 µM, while IC_50_ value for SiHa was 39 ± 4.07 µM, a 13-fold increase (Fig. 8).

## Discussion

Cancer cell lines are unique tools for understanding biology and tumor response. At present, immortalized cervical cancer cell lines available in major cell repositories, including ATCC or ECACC, represents only six cervical cancers (not considering HeLa-derived cell lines), not reflecting the whole spectrum of cervical cancer biological heterogeneity, neither representing patients from South America.

In the present study, we successfully established a cervical cancer cell line, HCB-514, out of 35 attempts - a successful rate of 3%. It is difficult to establish cervical cell lines, with success rates ranging from 11–30%, which may explain the reduced number of new cell lines^9–12^.

Not all mechanisms of continuous cell growth *in vitro* are known, but, at least for HPV-positive cervical cancers, the presence of papillomavirus E6/E7 expression is an important requirement^12^. Importantly, the HCB-514 cell line was HPV-16 positive and expressed E6 oncoprotein. Moreover, in our cell line and respective primary tumor, we showed that HPV integrates into the *CDH13* gene. This gene negatively regulates keratinocyte proliferation and has been reported as one of the integration-targeted cellular genes (ITGs) in cervical cancer^13^. Although the role of these ITGs in carcinogenesis is not completely understood, it is suggested that ITGs modulate the expression of nearby genes through a long-distance chromatin interaction mechanism^13^. In addition, we used phylogenetic analysis to confirm the HPV16 A1 variant in both HCB-514 and primary tumor. This variant has been correlated with a high risk of precancer and cancer in Caucasian women^14^.

The molecular characterization of the HCB-514 cell line showed mutations in important cancer-related genes, yet exhibited a distinct profile compared to the profile for cervical squamous cell carcinomas recently reported by TCGA^4^. One of the mutated genes, *BRCA2*, is a well-known hereditary breast cancer gene that encodes a protein related to DNA damage repair during homologous recombination^15^. Mutations in *BRCA2* occur in approximately 3% of cervical cancers according to the TCGA dataset www.cbioportal.org. Notably, there is limited knowledge of *BRCA2* mutational status in cervical cancer, and current literature focuses on germline mutations and its role on non-HPV related cervical cancers, mostly associated with hereditary syndromes^16,17^. There are no functional studies about *BRCA2* mutation in cervical cancer. Interestingly, the pathogenic p.W2830* *BRCA2* mutation identified in HCB-514 has been identified in other cancers as a predictive marker for targeted therapies, such as PARP inhibitors; its role in cervical cancers remains unclear.

We also observed a mutation in the *TGFBR1* gene, which encodes a receptor that acts on the TGF-β signaling pathway. This pathway is also related to the *TGFBR2* gene, which is one of the most commonly mutated genes in cervical cancer^4^. When TGBR2 is activated by a TGF-β ligand, TGBR1, a serine/threonine kinase, is dimerized, leading to the cell cycle arrest of epithelial cells, control of mesenchymal cell proliferation and differentiation, and other cell processes related to growth suppression^18^. In this sense, TGF-β inhibitors, such as galunisertib, may emerge as a new approach for the treatment of cervical cancer in *TGFBR1-*mutated tumors^19^.

We also observed the presence of a mutation in the *IRX2* gene. The *IRX2* belongs to the iroquois homeobox gene family and encodes a transcription factor known to play multiple roles during embryo development^20^. In breast cancer, low expression of *IRX2* has been observed and shown to be associated with the presence of disseminated tumor cells^21^. In this line, cell lines edited to overexpress IRX2 exhibited lower cellular motility and reduced secretion of pro-metastatic chemokines^20^. Moreover, the methylation of the *IRX2* promoter region has been correlated with pleural anthracosis disease in lung adenocarcinomas^22^. Controversially, in primary human osteosarcoma tissues, *IRX2* expression was observed to be significantly increased in comparison with normal tissues, and was significantly associated with tumor progression and prognosis^23^. *In vitro* assays showed that IRX2 upregulated MMP-9 and VEGF in a PI3K/AKT-dependent manner, and that knockdown of IRX2 in osteosarcoma cell lines inhibited cell proliferation and invasion^23,24^.

Another gene of interest that was mutated in both the HCB-514 cells and patient’s tumor tissue was *SHKBP1*. Recently, *SHKBP1* was identified in the cervical cancer TCGA, as a significantly mutated gene in this tumor type^4^. Overexpression of SHKBP1 in cell lines led to a strong delay in the degradation of EGFR upon EGF activation^25^. This process occurred due to competitive binding of SHKBP1 with protein c-Cbl, thereby interrupting the complex c-Cbl-CIN85, which is responsible for endocytosis of EGFR containing vesicles^25^.

According to TCGA, an average of 88 somatic CNV was found per cervical tumor, including 26 amplifications, 37 deletions and 26 alterations in whole chromosome arms^4^. The HCB-514 cell line presented 30 amplifications and 34 deletions, showing 64 alterations in total. Among these CNVs, *EGFR* and *YAP1* genes were amplified in the HCB-514 cell line. Both amplifications have already been described as recurrent in cervical cancer^4^. In addition, to confirm the biological impact of *EGFR* gene amplification we analyzed and observed EGFR protein phosphorylation in the HCB-514 cell line. Interestingly, blocking the EGFR tyrosine-kinase protein is an effective approach to treat specific types of tumors, including colorectal, head and neck and lung cancers^26^.

In cervical cancer, a phase II clinical study with EGFR inhibitor Erlotinib showed that administering it prior to receiving the standard of treatment (cisplatin concurrent with radiotherapy) was safe and showed significant antitumor activity^27^. In addition, a recent study demonstrated a promising effect of lapatinib (anti- EGFR and HER-2), in HER-expressing cervical cancer cells by reducing the number and size of blood vessels and preventing increased HIF-1α levels^28^. Regarding YAP1, the protein encoded by *YAP1* gene, it is negatively regulated by the Hippo signaling pathway, and its enhanced activity is believed to induce cell proliferation, migration and survival in several cancer types including cervical cancer^29,30^. Increased activity of YAP1 can occur after TGFα and amphiregulin inhibit the Hippo signaling via EGFR^29^. Furthermore, *YAP1* seems to play a key role in TGF-β1-induced apoptosis and EMT through EGFR regulation^30^. In cervical cancer, *YAP1* was amplified in the EMT cluster samples from TCGA data highlighting the potential role of this gene in EMT-related tumor progression^4^. However, more functional studies on YAP1 amplification need to be conducted to elucidate its role in cervical cancer.

Finally, we showed the sensitivity of HCB-514 to cisplatin, in line with the patient’s clinical response to cisplatin (with concurrent radiotherapy).

In conclusion, the molecular profile of the novel Brazilian cervical cancer cell line HCB-514 reveals disruption of several key cellular pathways, such as PI3K-AKT, MAPK signaling, DNA repair, apoptosis, transcriptional factors and RTK. This new HPV-16 positive cervical cancer cell line constitutes an important model to better understand the cancer biology of cervical cancer and offers an additional promising tool for cervical cancer pre-clinical studies.

## Materials and Methods

### Establishment of cervical cancer cell lines

Blood and tumor tissue samples were collected from participants with cervical cancer during routine visits in the Gynecologic Oncology Department of BCH after informed consent was obtained. The collected tumor tissue was maintained in PBS buffer in the refrigerator until processing, which occurred before 24 hours. The sample was initially placed in a petri dish and fragmented with scalpels and incubated for 30 min at 37 °C in a 15 mL falcon containing 5 mL of an enzymatic solution (0.14% collagenase type I [Sigma, Brazil] and 0.01% DNase [2,000 kilounits/mg] [Sigma]) in RPMI 1640 as previously described^31^. Then, RPMI medium was supplemented with 1% penicillin and streptomycin (P/S; Sigma) and 10% fetal bovine serum (FBS; Sigma) was added to inactivate the enzyme. After centrifugation, the cell pellet was resuspended in FAD media^32^ adapted by Dr. Matias Melendez (personal communication), which was comprised by three parts of Dulbecco’s modified Eagle’s media (DMEM; Sigma) to one part Ham’s F12 media (F12; Sigma). The media was then supplemented with 1% P/S, 0.4 µg/mL hydrocortisone, 2.5 µg/mL insulin, 13 ng/mL liothyronine, 5 µg/mL transferrin, 2.5 µg/mL insulin and 0.1 µg/mL cholera enterotoxin and 5% FBS (all supplements were purchased from Sigma). Cells were maintained in a T25 flask at 37 °C, 5% CO_2_. One week after processing, the media was replaced and cell growth was monitored. Controlled trypsinizations were done to preferentially remove the contaminating fibroblasts. Afterwards, the culture media was replaced every 72 h and the cell culture was routinely subcultured once it was confluent. Stock vials were frozen in FBS+ 5% DMSO solution for storage in liquid nitrogen. All methods were performed in accordance with the relevant guidelines and regulations and were approved by the Ethics and Research Committee from Barretos Cancer Hospital (BCH) (985/2015 - CAAE 1,252,699).

### Cell lines

For molecular and functional characterization, the immortalized cervical squamous cell carcinoma cell lines, SiHa, was kindly provided by Dr. Luisa Villa (INCT-HPV, Brazil) and a human short-term primary culture of lung fibroblast, named HCB-535, was obtained from BCH. SiHa was grown in DMEM media supplemented with 1% P/S and 10% FBS and HCB-535 in RPMI media supplemented with 1% P/S and 10% FBS.

### Immunophenotypic characterization

To confirm cell origin, immunocytochemistry was performed with antibodies against vimentin (positive for fibroblasts) and cytokeratin (positive for epithelial cells). When cells reached the fourth passage, they were plated in a 24-well plate (5 × 10^5^ in 3 DMEM:1 F12 media + 5% FBS) and, upon reaching 80% confluence, cells were fixed with 4% paraformaldehyde for 15 min, washed 3 times with PBS, permeabilized with Triton X-100 (0.1% in water) for 4 min and washed as described. Endogenous peroxidase activity was inhibited for 10 min with H_2_O_2_ (3% in methanol). Blockage for non-specific labeling was performed through incubation with 50 µL of the Ultra V Block reagent (Thermo Scientific detection kit) for 10 min and, after washes, primary antibodies cytokeratin AE1/AE3 (Dako, ready to use) or vimentin (Dako, ready to use), incubation was done for 60 min and then washed. A biotinylated goat polyvalent antibody incubation for 10 min was performed, followed by washing. Streptavidin peroxidase incubation was next performed for 10 min and washed. Afterwards, the DAB chromogen (ThermoFisher Scientific) was applied for 10 min for staining. Finally, hematoxylin was used for counterstaining and the cells were photographed with a photographic camera coupled to a microscope (Olympus). The immortalized cell line SiHa, a grade II squamous cell carcinoma and HPV-16 positive, was used as a positive control for the cytokeratin and HCB-535 was used as positive control for vimentin.

The markers p40 (Biocare Medical, dilution 1:100) and p16 (Roche, Ventana Systems, ready to use), commonly used in the routine pathology for determination of cervical cancer histology subtype^33^, were evaluated by immunohistochemistry in the cell block of the new cervical cancer cell line established in this work and in FFPE tumor tissue from the cervical cancer patient. Moreover, expression of EpCAM (CD326, BD Biosciences)^34^, another marker of cervical carcinoma cells, was analyzed in the HCB-514 cell line by flow cytometry. Briefly, 5 × 10^5^ cells were harvested, washed twice in BSA 0,2% in DPBS (centrifugation between the washes was 1500 rpm for 5 min), then 10 µL of EpCAM PE-conjugate were added and incubated for 20 min. Afterwards, cells were washed and analyzed by flow cytometry (Accuri BD). SiHa was used as positive control for EpCAM expression and HCB-535 was used as negative control.

### Cell line doubling-time

Growth characteristics of the cell line were analyzed by doubling-time evaluation. Doubling-time was determined through real time impedance-based technology cell analyzer system (RTCA, xCELLigence, Roche), according to manufacturer’s instructions. Firstly, 5,000 cells were plated into E-plate (xCELLigence, Roche) and proliferation rate was evaluated for 72 h. The new cervical cancer was maintained in supplemented media above described in establishment section, with 5% FBS or 10% FBS. SiHa was maintained in DMEM 1% P/S and 5 or 10% FBS. This assay results in a cell index, which considers cell viability, cell number, morphology and adhesion. At the end of assay, a cell growth curve is produced and the doubling-time was calculated from the selected interval, which is basically the interval selected when the cell index doubled inside the log-phase^35^.

### DNA isolation

For molecular characterization, DNA was isolated from 1 × 10^6^ tumor cell pellet in the sixth passage using silica column from Biopur Mini Spin Plus 250 Extraction kit (Biopur), as described by the manufacturer. Blood total DNA was automatically isolated from buffy coat of patient through Mini Kit DNA and Qiasymphony instrument (both from Qiagen) at BCH Biobank Department, according to the manufacturer. This technology combines silica-based extraction with magnetic particles purification. The protocol Blood 200 with an elution volume of 50 µL was used. Tumor DNA was isolated from a biopsy sample obtained from the patient through the Mini Kit DNA and Qiasymphony instrument (QIAgen). For tumor-isolated DNA, macrodissection was performed by an experienced pathologist and up to 25 mg of the sample containing at least 60% tumor area and up to 20% necrosis area were fragmented and submitted to digestion and homogenization procedures, according to the manufacturer’s instructions. The protocol Tissue 200 with an elution volume of 50 µL was used.

All DNA samples were quantified by both NanoDrop® 2000 (Thermo Scientific) and Qubit® 2.0 Fluorometer (Life Technologies) and were then stored at −20 °C for further genetic analysis. Blood DNA was used as reference to distinguish somatic from germline mutations.

### Assessment of human papilloma virus (HPV)

The presence of HPV in HCB-514 cell line was evaluated by in house PCR using GP5+ and GP6+ primers, able to amplify a fragment of ~142 pb of the HPV conserved region L1^36^. For this, it was used 5 µL of DNA extracted from HCB-514, 20 mM of Thris-HCl buffer (pH 8.4), 50 mM KCl, 1.25 U of Platinum Taq DNA polymerase, 2 mM of MgCl2, 0.2 mM dNTP mix, 0.6 µM of each primer (GP5+ and GP6+). Amplifications were performed in the equipment Applied Biosystems GeneAmp PCR System 2700 (Applied Biosystems) and the cycle was as described: 94 °C for 4 min followed by 40 cycles of 1 min at 94 °C, 2 min at 40 °C, and 1 min 30 seg at 72 °C. Then, a cycle of 72 °C for 7 min and the PCR products were stored at 4 °C before electrophoresis on a 2% agarose gel. DNA HPV-positive was used as positive control and water was used as negative control. PCR reagents were pursued from Invitrogen.

Moreover, the COBAS® 4800 HPV System (Roche) was used for genotyping HPV, according with manufacturer’s instruction. This assay utilizes DNA target amplification through real time PCR to detect 14 high-risk HPVs in a single assay. It genotypes HPVs 16 and 18 and simultaneously detects other non-16 and 18 high-risk types (31, 33, 35, 39, 45, 51, 52, 56, 58, 59, 66 and 68) as a pool result in cases of infection.

In addition, because E6 oncoprotein is required for the oncogenic transformation of HPV-infected cervical epithelial cells and the most common high-risk HPVs associated with cervical cancer are 16 and 18, the presence of their E6 oncoprotein was assessed through an immunochromatographic test, using OncoE6^TM^ Cervical Test (Arbor Vita), according with manufacturer’s instructions. Briefly, the lysate of 1 × 10^6^ HCB-514 cells was incubated with monoclonal antibodies to oncoprotein E6 of HPV types 16 and 18 conjugated with alkaline phosphatase. Then, using a nitrocellulose test strip, this mix migrates up the test strip and if the protein is present, it is possible to observe a purple line in the strip. The position of the line indicates if E6 is from HPV-16 or HPV-18.

### Cell line authentication by short tandem repeat (STR) profiling analysis

The STR analysis was carried out in DNA from cell culture, FFPE tumor tissue and blood from the patient as previously described for authenticity confirmation^37,38^.

### Whole exome sequencing (WES) analysis

The DNA from the HCB-514 cell line, the patient’s primary tumor and paired blood sample were used for WES, with input of 50 ng on the Illumina HiSeq2500 ™ System by a commercial company (Mendelics, São Paulo, Brazil). Sequence reads were aligned to the human reference genome build 37 (hs37d5-decoy) using BWA-MEM with Burrows–Wheeler Aligner version 0.7.10-r789^39^. Duplicate reads were marked with Picard-Tools 1.92 (http://broadinstitute.github.io/picard/). MuTect version 1.1.4; (http://www.broadinstitute.org/cancer/cga/mutect) and Varscan2^40^ were used to call somatic SNVs and indels in tumor-normal pairs, respectively. MuTect was run using default parameters with files from COSMIC version 54 and dbSNP version 132 included as input^41,42^. We used Ensembl Variant Effect Predictor (VEP)^43^ to annotate and determine functional consequences of tumor specific variants. The results from SIFT, Polyphen-2, ClinVar were considered. It was also excluded variants that were likely to be germline, i.e., listed in ESP6500 (http://evs.gs.washington.edu/EVS/), 1000Genome or ExAC^44,45^. The candidate mutations were validated visually using the Integrated Genomics Viewer (IGV)^46^. Copy number abnormalities (CNA) were identified using Nexus Copy Number version 9.0 (BioDiscovery; El Segundo, CA; https://www.biodiscovery.com/products/Nexus-Copy-Number) with default parameter for BAM ngCGH (matched) input with homozygous frequency threshold and value at 0.97 and 0.8 respectively, hemizygous loss threshold at −0.18, single copy gain at 0.18 and high copy gain at 0.6.

Mutational signature was defined using the package SomaticSignatures of Bioconductor Software, as described by Gehring *et al*.^47^.

Integration of HPV viral DNA into the human genome was performed through integration mode of the program HPVDetector, as previously described^48^. The phylogenetic analysis of HPV sequence was performed using the fasta consensus sequences of HPV16 (one from tumor sample and the other from cell culture) obtained from deep sequencing and a group of HPV16 complete genome sequences retrieved from GenBank were aligned with Muscle^49^ and edited with Se-Al v2.0a11 (available at http://tree.bio.ed.ac.uk/software/sea/). Phylogenetic reconstructions were performed by maximum likelihood (ML) criterion using RAxML version 8.0.0^50^. The best fit model used for the ML reconstruction was GTR + Γ model without partitions. Node support was evaluated using 1,000 bootstraps cycles.

### *BRCA2* Sanger sequencing

To validate the mutation identified in WES in *BRCA2*, the HCB-514 cell and patient’s blood DNAs were submitted to PCR amplification of *BRCA2* exon 20, using Hot Start Taq enzyme (Qiagen), as described by Costa *et al*.^51^. After amplification, PCR products were purified with the ExoSap enzyme (USB Affymetrix), and submitted to sequencing protocol using BigDye Terminator v3.1 Cycle Sequencing Kit (Applied Biosystems). Sequencing was performed in 3500 XL Genetic Analyzer (Applied Biosystems), as described by Fernandes *et al*.^52^.

### Copy number variation analysis

HCB-514 chromosomal alterations were also analyzed by the Nanostring plataform, using the nCounter® v2 Cancer CN Assay panel (NanoString Technologies, Seattle, WA, USA). This panel counts the CNV of 87 involved genes commonly amplified or deleted in various cancers (www.nanostring.com/products/CNV). As control, the genomic DNA from the HCB-514 patient’s peripheral blood was used. The DNA input was 600 ng for HCB-514, and 473 ng for peripheral blood.

The raw data was captured by the nSolverAnalysis Software v3.0® program (NanoString Technologies). For the normalization of the results, 54 probes were used for regions of the genome that do not usually present CNV. After the data normalization, the mean of the counts from the 3 probes per each gene was calculated and the number of copies per gene was calculated as previously described^53,54^.

### Human-receptor tyrosine kinase (RTK) array and *Western blot*

To characterize HCB-514 protein expression, the Proteome Profiler Human Phospho-RTK Array Kit (#ARY001, R&DSystems) was used, according to the manufacturer’s instructions, and basal and epidermal growth factor (EGF)-stimulated protein expression were evaluated. Briefly, cells were grown in two T75 flask until confluence. Then, for EGFR stimulation, EGF diluted in 10 mL of free-serum media (final concentration 10 ng/mL) was firstly added to the cells and incubated for 15 min. After, proteins were collected using lysis buffer (50 mM Tris pH7.6–8, 150 mM, NaCl, 5 mM EDTA, 1 mM Na3VO4, 10 mM NaF, 10 mM,sodium pyrophosphate, 1% NP-40, and protease cocktail inhibitors) and 500 µg of protein lysates were incubated as previously described^55^. Membranes were revealed with ECL Western Blotting Detection Reagents (RPN2109, GE Healthcare) and the chemiluminescent signal was detected by ImageQuant™ LAS 4000 mini documentation system (GE Healthcare).

For *Western blot*, protein lysates were separated in 8% SDS-PAGE gel, transferred, incubated in phospho-EGFR^Y1068^ (1:1000), EGFR (1:1000) or α-tubulin (1:2000) primary antibody and revealed, as detailed described by Silva-Oliveira *et al*.^56^. Antibodies were purchased from Cell Signaling company.

### Viability evaluation

The cell viability of the HCB-514 cell line to cisplatin was evaluated by MTS assay as previously reported^38^. Briefly, 6,000 cells were plated in 96-wells, allowed to adhere, and then treated with a range from 0.01 to 8 µM of cisplatin (Sigma). After 72 h, the Cell Titer 96 Aqueous Cell Proliferation Assay (MTS reagent, Promega) was added and incubated for 3 h. Absorbance values were normalized with absorbance of DMSO 1%-treated cells and the IC_50_ (inhibitory concentration for 50% cells) was calculated through non-linear regression.

### Statistical analysis

GraphPad Prism was used to determine IC_50_ in drug-response assays and to plot the cell index data from doubling-time assay and the relative protein expression from *Western blot* analysis.

## Supplementary information

Supplementary material

## Data Availability

Genetic data has been deposited at the European Genome-phenome Archive (EGA, http://wwwdev.ebi.ac.uk/ega/), which is hosted by the EBI, under accession number EGAS00001003343.
