# Supplementary material for: Establishment, molecular and biological characterization of HCB-514: a novel human cervical cancer cell line
Source: Sci Rep. 2019 Feb 13;9:1913. doi: 10.1038/s41598-018-38315-7 (PMC6374403; doi:10.1038/s41598-018-38315-7)
Supplement: Supplementary file 1 — Supplementary material [file 41598_2018_38315_MOESM1_ESM.docx]

# Establishment, molecular and biological characterization of HCB-514: a novel human cervical cancer cell line.

Marcela Nunes Rosa^1^; Adriane Feijó Evangelista^1^; Letícia Ferro Leal^1^; Cristina Mendes de Oliveira^1^, Viviane Aline Oliveira Silva^1^; Carla Carolina Munari^1^; Fernanda Franco Munari^1^; Graziela de Macêdo Matsushita^2^; Ricardo dos Reis^3^; Carlos Eduardo Andrade^3^; Cristiano de Pádua Souza^3^; Rui Manuel Reis*^1,4,5^

^1^Molecular Oncology Research Center; ^2^Department of Pathology; ^3^Department of Gynecologic Oncology, Barretos Cancer Hospital, Barretos, SP, Brazil; ^4^Life and Health Sciences Research Institute (ICVS), School of Health Sciences, University of Minho, Braga, Portugal; ^5^3B's—PT Government Associate Laboratory, Braga/Guimarães, Portugal.

# *Corresponding author:

# Rui Manuel Reis, PhD

# Molecular Oncology Research Center, Barretos Cancer Hospital

# Rua Antenor Duarte Villela, 1331

# CEP 14784 400, Barretos, S. Paulo, Brazil

# Email: ruireis.hcb@gmail.com

# Telephone:+551733216600

**Supplementary table S1**. Clinic-pathological characteristics of patients.

|  | **Cell line** | **Age** | **Histology** |
| --- | --- | --- | --- |
| 1 | HCB-483 | 46 | SCC |
| 2 | HCB-484 | 58 | SCC |
|  | HCB-485 | 43 | SCC |
| 3 | HCB-486 | 51 | SCC |
| 4 | HCB-487 | 73 | SSC |
| 5 | HCB-488 | 44 | SCC |
| 6 | HCB-489 | 60 | SCC |
| 7 | HCB-490 | 65 | SCC |
| 8 | HCB-491 | 47 | SCC |
| 9 | HCB-492 | 55 | SCC |
| 10 | HCB-493 | 49 | Adenocarcinoma |
| 11 | HCB-494 | 68 | Adenocarcinoma |
| 12 | HCB-495 | 26 | SCC |
| 13 | HCB-496 | 68 | SCC |
| 14 | HCB-497 | 82 | SCC |
| 15 | HCB-498 | 59 | SCC |
| 16 | HCB-499 | 33 | SCC |
| 17 | HCB-500 | 70 | Adenosquamous |
| 18 | HCB-501 | 30 | SCC |
| 19 | HCB-502 | 49 | SCC |
| 20 | HCB-503 | 93 | SCC |
| 21 | HCB-506 | 65 | SCC |
| 22 | HCB-507 | 48 | SCC |
| 23 | HCB-508 | 40 | SCC |
| 24 | HCB-510 | 43 | SCC |
| 25 | HCB-512 | 80 | SCC |
| 26 | HCB-513 | 63 | Adenocarcinoma |
| 27 | HCB-514 | 30 | SCC |
| 28 | HCB-518 | 35 | SCC |
| 29 | HCB-522 | 53 | SCC |
| 30 | HCB-523 | 35 | SCC |
| 31 | HCB-524 | 58 | SCC |
| 32 | HCB-526 | 57 | SCC |
| 33 | HCB-537 | 75 | SCC |
| 34 | HCB-538 | 25 | SCC |
| 36 | HCB-542 | 42 | SCC |
| 37 | HCB-546 | 35 | SCC |

**Supplementary table S2.** Total 93 non-silent mutations found in HCB-514.

| **Chromosome** | **Position** | **Gene** | **Referrence allele** | **Variant**  **allele** | **Aminoacid** |
| --- | --- | --- | --- | --- | --- |
| 1 | 17185537 | RP11-108M9.2 | G | T | - |
| 1 | 17930009 | ARHGEF10L | G | C | p.R119P |
| 1 | 72058537 | NEGR1 | G | T | p.N301K |
| 1 | 109560148 | WDR47 | G | T | p.D78E |
| 1 | 145818808 | GPR89A | C | A | p.G21W |
| 1 | 225328450 | DNAH14 | A | T | p.N1395I |
| 1 | 228335177 | GUK1 | C | T | p.R129W |
| 2 | 43817966 | THADA | G | T | p.A100E |
| 2 | 69049563 | ARHGAP25 | G | A | p.G431E |
| 2 | 96521280 | ANKRD36C | T | G | p.I1577L |
| 3 | 21706378 | ZNF385D | C | T | p.A55A |
| 3 | 27763616 | EOMES | C | T | p.G57D |
| 3 | 77657079 | ROBO2 | C | A | p.H1105Q |
| 3 | 124420944 | KALRN | G | A | p.E989K |
| 3 | 125032193 | ZNF148 | C | T | p.E98K |
| 3 | 148875136 | HPS3 | G | T | p.X504_splice |
| 3 | 167000256 | ZBBX | G | C | p.A675G |
| 3 | 193039604 | ATP13A5 | G | C | p.T594S |
| 3 | 197249504 | BDH1 | G | A | - |
| 5 | 2751384 | IRX2 | G | T | p.Y48* |
| 5 | 5489376 | KIAA0947 | G | C | p.S2245T |
| 5 | 14713075 | ANKH | C | A | p.G425C |
| 5 | 35010154 | AGXT2 | T | G | p.D430A |
| 5 | 36136508 | LMBRD2 | G | T | p.A217E |
| 5 | 140793112 | PCDHGA10 | G | A | p.E124K |
| 6 | 26501990 | BTN1A1 | A | T | p.E84D |
| 6 | 43528041 | XPO5 | C | A | p.X366_splice |
| 6 | 159660971 | FNDC1 | G | A | p.E1535K |
| 7 | 33945363 | BMPER | G | T | - |
| 7 | 77408339 | RSBN1L | A | C | p.K799Q |
| 8 | 120594668 | ENPP2 | A | G | p.V625A |
| 8 | 142367558 | GPR20 | C | T | p.E156K |
| 8 | 144809668 | FAM83H | C | T | p.E655K |
| 9 | 156528 | CBWD1 | C | A | p.X177_splice |
| 9 | 101900171 | TGFBR1 | C | T | p.A202V |
| 10 | 93694 | TUBB8 | T | C | p.K213R |
| 10 | 16867082 | CUBN | C | A | p.X3589_splice |
| 10 | 19620301 | MALRD1 | G | T | p.V540F |
| 10 | 51130414 | PARG | G | A | p.L59F |
| 10 | 61844594 | ANK3 | C | A |  |
| 10 | 102058330 | PKD2L1 | G | C | p.F240L |
| 10 | 121432151 | BAG3 | G | A | p.V298M |
| 11 | 28057764 | KIF18A | C | T | p.R799Q |
| 11 | 108384207 | EXPH5 | C | T | p.S676N |
| 12 | 49087267 | CCNT1 | G | A | p.S577F |
| 12 | 72956810 | TRHDE | C | T | p.Q633* |
| 12 | 99194813 | ANKS1B | C | G | p.E1053Q |
| 12 | 109201563 | SSH1 | G | A | p.R193W |
| 13 | 32945095 | BRCA2 | G | A | p.W2830* |
| 14 | 22447320 | TRAV8-6 | C | T | p.H101Y |
| 14 | 75618762 | TMED10 | T | C | p.M104V |
| 15 | 23406819 | RP11-467N20.5 | T | C | p.K673E |
| 15 | 39874463 | THBS1 | G | A | p.R46H |
| 15 | 43545745 | TGM5 | G | A | p.R215W |
| 15 | 74710316 | SEMA7A | C | T | - |
| 16 | 24942731 | ARHGAP17 | C | A | - |
| 16 | 71212844 | HYDIN | C | A | p.R123M |
| 16 | 72162648 | PMFBP1 | G | A | p.R666* |
| 16 | 89347621 | ANKRD11 | G | A | p.Q1777* |
| 17 | 1559700 | PRPF8 | T | C | p.I1927V |
| 17 | 19289664 | MFAP4 | G | A | p.P91S |
| 17 | 19702886 | ULK2 | C | A | p.G482C |
| 17 | 26729306 | SLC46A1 | G | A | p.T372I |
| 17 | 36894999 | PCGF2 | G | A | - |
| 17 | 72352995 | BTBD17 | A | G | p.V413A |
| 17 | 74622837 | ST6GALNAC1 | C | T | p.D403N |
| 18 | 21042924 | RIOK3 | A | T | - |
| 18 | 48255712 | MAPK4 | C | T | p.R418C |
| 19 | 1880950 | ABHD17A | T | C | p.K144E |
| 19 | 13259847 | STX10 | C | T | p.R120K |
| 19 | 14910434 | OR7C1 | A | C | p.M172R |
| 19 | 15852363 | OR10H3 | G | A | p.R54H |
| 19 | 41084418 | SHKBP1 | G | A | p.V124I |
| 19 | 50412217 | NUP62 | C | G | p.S283T |
| 19 | 50463982 | SIGLEC11 | T | G | p.E96A |
| 19 | 52327351 | FPR3 | C | T | p.T117I |
| 19 | 55570646 | RDH13 | G | C | - |
| 20 | 4163484 | SMOX | G | A | p.R453H |
| 20 | 23066708 | CD93 | G | A | p.S41L |
| 21 | 47421214 | COL6A1 | G | C | p.E624Q |
| 22 | 17590484 | IL17RA | C | G | p.S792C |
| 22 | 19213831 | CLTCL1 | C | T | p.A620T |
| 22 | 25573381 | KIAA1671 | C | T | p.R1656W |
| 22 | 45574274 | NUP50 | T | A | p.S166T |
| X | 8138258 | VCX2 | G | T | p.Q79K |
| X | 11793095 | MSL3 | C | T | - |
| X | 31187708 | DMD | G | T | p.H3469N |
| X | 49105196 | CCDC22 | G | T | p.X477_splice |
| X | 71788696 | HDAC8 | G | A | p.A68V |
| X | 100653041 | GLA | C | A | p.W349L |
| X | 111698846 | ZCCHC16 | G | T | p.R297L |
| X | 123556182 | TENM1 | T | A | p.I1471F |
| X | 123556192 | TENM1 | C | A | p.E1467D |

**Supplementary table S3.** Total 68 non-silent mutations found in tumor.

| **Chromosome** | **Position** | **Gene** | **Referrence allele** | **Variant**  **allele** | **Aminoacid** |
| --- | --- | --- | --- | --- | --- |
| 1 | 108679461 | SLC25A24 | T | A | p.X417_splice |
| 1 | 219385056 | LYPLAL1 | G | A | p.E234K |
| 1 | 225328450 | DNAH14 | A | T | p.N1395I |
| 1 | 226075708 | LEFTY1 | A | G | p.L92S |
| 1 | 228335177 | GUK1 | C | T | p.R129W |
| 2 | 69049563 | ARHGAP25 | G | A | p.G431E |
| 2 | 89399780 | IGKV1-16 | A | C | p.C16W |
| 2 | 96688755 | GPAT2 | C | G | p.E722D |
| 2 | 96688768 | GPAT2 | A | G | p.L718S |
| 2 | 99785851 | MITD1 | G | T | p.T246K |
| 2 | 99785855 | MITD1 | G | T | p.H245N |
| 2 | 160303343 | BAZ2B | G | T | p.Q216K |
| 3 | 27763616 | EOMES | C | T | p.G57D |
| 3 | 37366231 | GOLGA4 | C | A | p.Q974K |
| 3 | 57389107 | DNAH12 | C | T | p.A2274T |
| 3 | 99569454 | FILIP1L | C | T | p.E356K |
| 3 | 99569459 | FILIP1L | A | T | p.I354K |
| 3 | 124420944 | KALRN | G | A | p.E989K |
| 3 | 133894464 | RYK | C | T | p.A523T |
| 3 | 135721101 | PPP2R3A | T | A | p.I254K |
| 3 | 169555405 | LRRIQ4 | G | A | p.G557R |
| 4 | 17585225 | LAP3 | C | A | p.Q167K |
| 4 | 23826133 | PPARGC1A | T | A | p.X253_splice |
| 4 | 114823502 | ARSJ | G | T | p.S576R |
| 4 | 146025639 | ABCE1 | C | A | p.C25* |
| 5 | 35010154 | AGXT2 | T | G | p.D430A |
| 5 | 78328665 | DMGDH | T | A | p.X455_splice |
| 5 | 140793112 | PCDHGA10 | G | A | p.E124K |
| 5 | 177580506 | NHP2 | G | T | p.N72K |
| 7 | 77408339 | RSBN1L | A | C | p.K799Q |
| 8 | 11659373 | RP11-297N6.4 | G | T | p.T75K |
| 8 | 49831529 | SNAI2 | C | A | p.C215F |
| 8 | 142367558 | GPR20 | C | T | p.E156K |
| 9 | 101900171 | TGFBR1 | C | T | p.A202V |
| 10 | 51130414 | PARG | G | A | p.L59F |
| 11 | 14889253 | PDE3B | G | A | p.G1030S |
| 11 | 64112607 | CCDC88B | G | A | p.R865Q |
| 12 | 38714080 | ALG10B | G | T | p.A163S |
| 12 | 49087267 | CCNT1 | G | A | p.S577F |
| 12 | 65462685 | WIF1 | C | A | p.X133_splice |
| 12 | 99194813 | ANKS1B | C | G | p.E1053Q |
| 12 | 109201563 | SSH1 | G | A | p.R193W |
| 13 | 32945095 | BRCA2 | G | A | p.W2830* |
| 13 | 41650357 | WBP4 | C | A | p.P247Q |
| 15 | 39874463 | THBS1 | G | A | p.R46H |
| 15 | 43545745 | TGM5 | G | A | p.R215W |
| 16 | 15457676 | NPIPA5 | A | G | p.L298P |
| 16 | 67237736 | ELMO3 | A | C | p.T760P |
| 16 | 72162648 | PMFBP1 | G | A | p.R666* |
| 17 | 26729306 | SLC46A1 | G | A | p.T372I |
| 17 | 56565403 | HSF5 | G | A | p.T78I |
| 17 | 72352995 | BTBD17 | A | G | p.V413A |
| 19 | 12575478 | ZNF709 | G | A | p.H420Y |
| 19 | 14910434 | OR7C1 | A | C | p.M172R |
| 19 | 41084418 | SHKBP1 | G | A | p.V124I |
| 19 | 52327351 | FPR3 | C | T | p.T117I |
| 19 | 53855879 | ZNF845 | C | G | p.Q651E |
| 20 | 4163484 | SMOX | G | A | p.R453H |
| 20 | 9546947 | PAK7 | G | A | p.Q359* |
| 20 | 23066708 | CD93 | G | A | p.S41L |
| 21 | 47421214 | COL6A1 | G | C | p.E624Q |
| 22 | 17590484 | IL17RA | C | G | p.S792C |
| 22 | 19213831 | CLTCL1 | C | T | p.A620T |
| X | 3228653 | MXRA5 | G | C | p.L2531V |
| X | 7811671 | VCX | C | A | p.Q79K |
| X | 18824609 | PPEF1 | G | A | p.R447Q |
| X | 49105196 | CCDC22 | G | T | p.X477_splice |
| X | 118975172 | UPF3B | C | T | p.R225K |

**Supplementary table S4.** Copy number variation regions in tumor.

| **Chromosome Region** | **CNV** | **Cytoband** | **Cancer genes** |
| --- | --- | --- | --- |
| chr1 | Deletion | p21.1 |  |
| **chr2** | **Amplification** | **p25.3 - p22.3** | ***MYCN, C2orf44, NCOA1, DNMT3A, ALK*** |
| chr2 | Homozygous deletion | p22.3 |  |
| chr3 | High amplification | q23 |  |
| **chr3** | **Deletion** | **q26.1** |  |
| **chr4** | **Deletion** | **p16.3 - p11** | ***FGFR3, WHSC1, SLC34A2, PHOX2B*** |
| chr4 | Deletion | q11 - q35.2 | *FIP1L1, PDGFRA, CHIC2, KIT, KDR, RAP1GDS1, TET2, IL2, FBXW7* |
| **chr5** | **Deletion** | **p15.33** |  |
| **chr5** | **Deletion** | **q13.2** |  |
| chr7 | Amplification | p11.2 |  |
| **chr7** | **Deletion** | **p11.2 - q36.3** | ***SBDS, ELN, HIP1, AKAP9, CDK6, MET, SMO, CREB3L2, KIAA1549, BRAF, EZH2, MLL3, SHH*** |
| **chr11** | **Deletion** | **q22.3 - q25** | ***ATM, DDX10, POU2AF1, SDHD, PAFAH1B2, PCSK7, MLL, DDX6, CBL, ARHGEF12, FLI1*** |
| **chr18** | **Deletion** | **p11.32 - p11.31** |  |
| **chr18** | **High amplification** | **p11.31** |  |
| chrX | Amplification | q23 |  |

In bold are alterations found also in HCB-514.


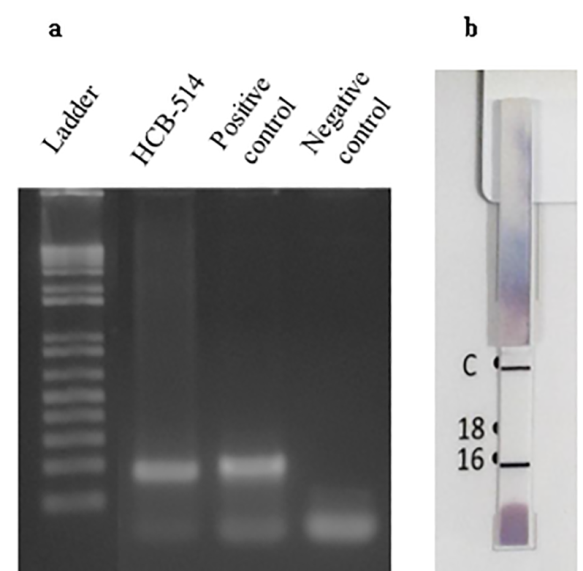


**Supplementary figure S1.** Detection of GP5+/GP6+ HPV after PCR by agarose gel electrophoresis (**a**) and OncoE6 protein test (**b**). Positive sample and HCB-514 bands show PCR products in 150 bp level.

**
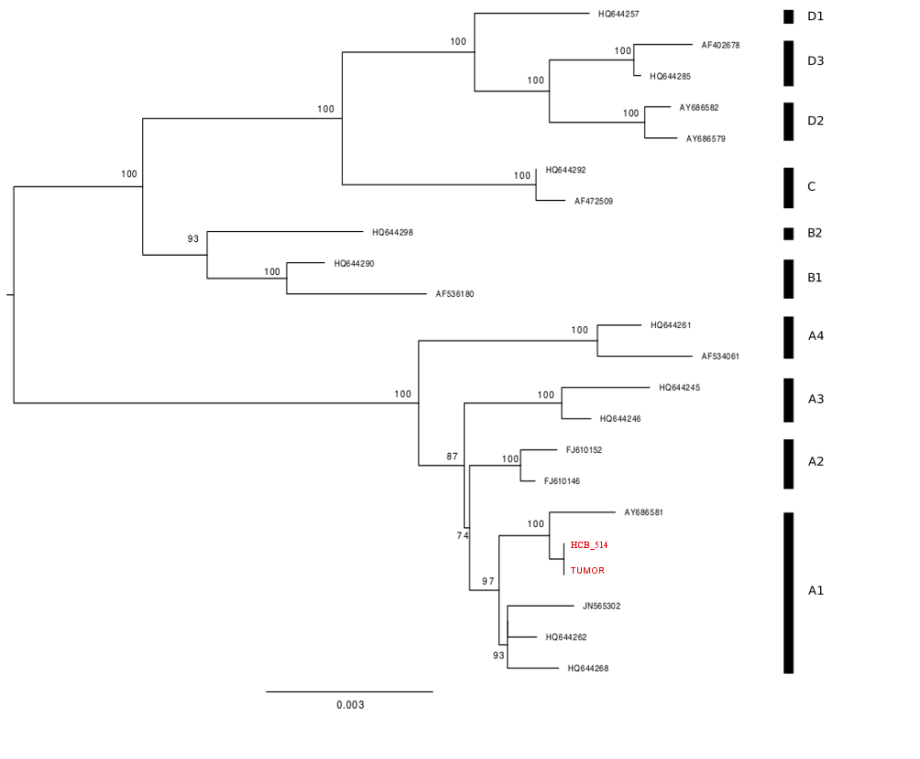
**

**Supplementary figure S2.** HPV16 complete genome unrooted maximum likelihood phylogenetic tree inferred with the study samples (red) and the HPV16 genome references using GTR+4Γ nucleotide substitution model. Support after 1000 bootstraps cycles is shown for each node.


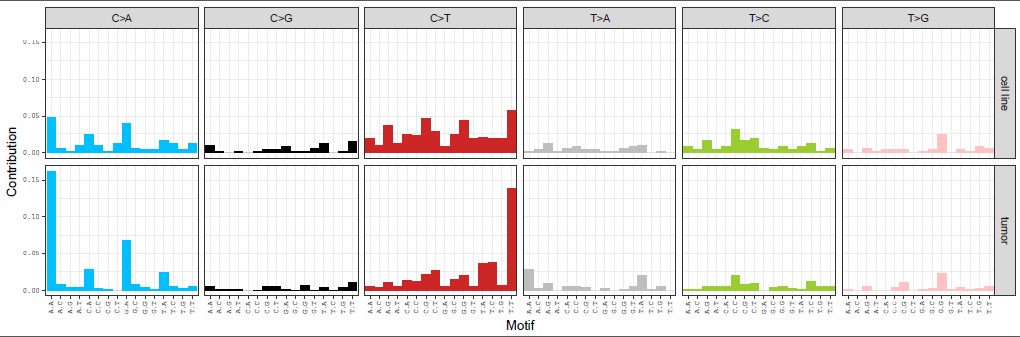


**Supplementary figure S3**. Mutational signature of HCB-514 cell line (top) and tumor (bottom) from WES data.

**
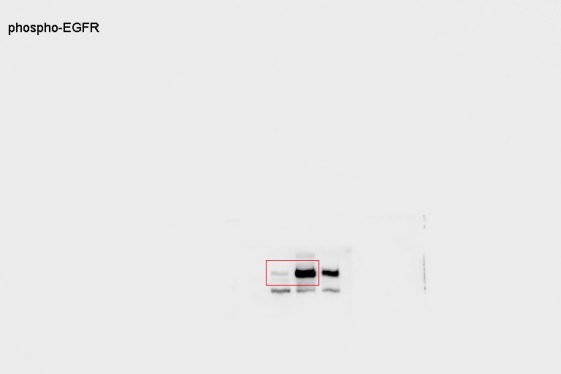

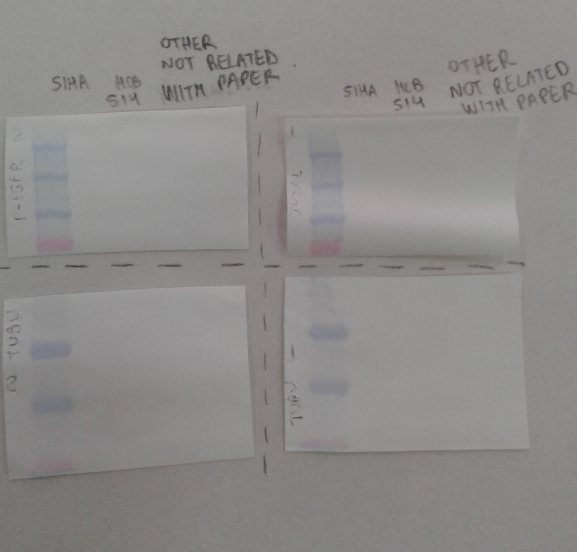
**

**
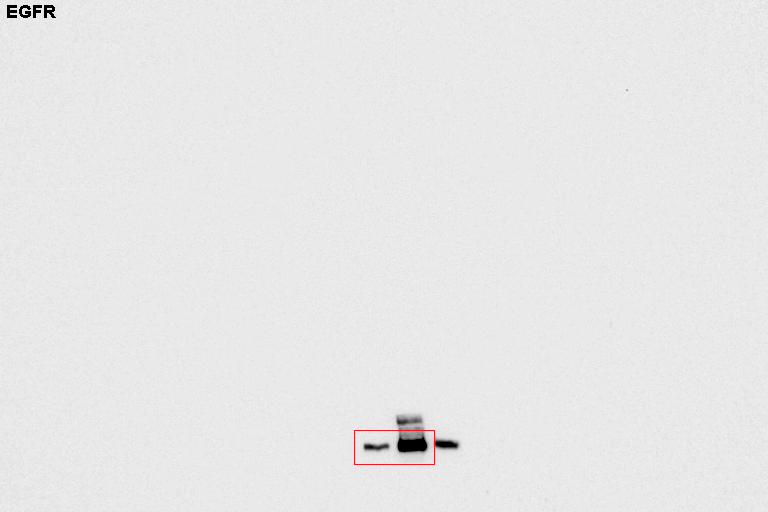
**

**
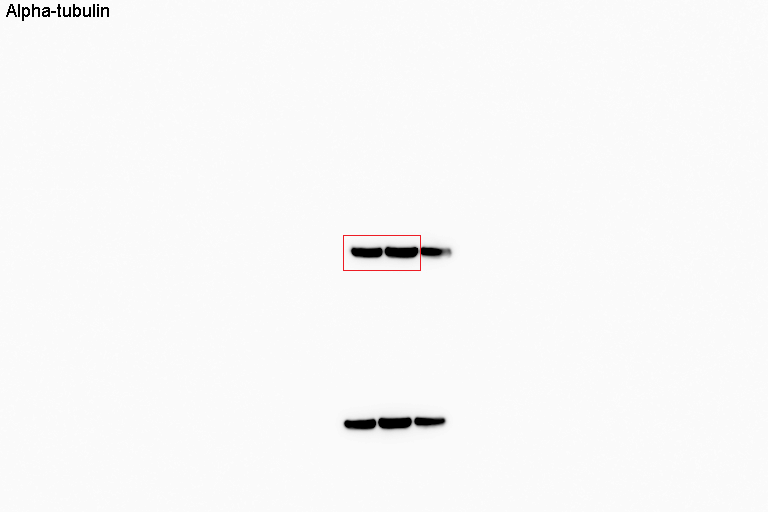
**

**Supplementary figure S4.** Ten-wells 8% SDS-PAGE gel was run with samples under the same experimental conditions. Picture of the cropped membrane showing the regions where it was cut for *Western blot* assay. Complete images obtained from chemiluminescent revelation are shown, with the region of interest for quantification highlighted.
